# Infant periodic breathing and apneic threshold

**DOI:** 10.14814/phy2.15915

**Published:** 2024-01-19

**Authors:** Stanley M. Yamashiro, Narayan P. Iyer

**Affiliations:** ^1^ Biomedical Engineering Department University of Southern California Los Angeles California USA; ^2^ Fetal and Neonatal Institute, Div of Neonatology, CHLA Dept of Pediatrics, Keck School of Medicine Los Angeles California USA

**Keywords:** apneic threshold, infants, periodic breathing

## Abstract

A mathematical model was proposed to predict the role played by apneic threshold in periodic breathing in preterm infants. Prior models have mainly applied linear control theory which predicted instability but could not explain sustained periodic breathing. Apneic threshold to CO2 which has been postulated to play a major role in infant periodic breathing is a nonlinear effect and cannot be described by linear theory. Another previously unexplored nonlinear factor affecting instability is brain vascular volume change with CO2 which affects time delay to chemoreceptors. The current model explored the influences of apneic threshold, central and peripheral chemoreceptor gains, cardiac output, lung volume, and circulatory time delay on periodic breathing. Apneic threshold was found to play a major role in ventilatory responses to spontaneous sighs. Sighs led to apneic pauses followed by periods of periodic breathing with peripheral chemoreceptor CO2 gain, cardiac output, and lung volume were at reported normal levels. Apneic threshold when exceeded was observed to cause an asymmetry in the periodic breathing cycling and an increased periodic breathing frequency. Sighs in infants occur frequently enough to lead to repeated stimulation within the epoch duration of periodic breathing for a single sigh. Multiple sighs may then play a major role in promoting continuous periodic breathing in infants. Peripheral chemoreceptor gain estimated using endogenous CO2 led to validated predicted periodic breathing cycle duration as a function of age. Brain vascular volume increase with CO2 contributes to periodic breathing in very young (1–2 day old) preterm infants.

## 1 INTRODUCTION

Periodic breathing has been reported to have a prevalence of 94.5% in low (<2500 g) and 36.1% in normal birth weight preterm infants (Fenner et al., 1973). Periods of apnea were 5–10 s followed by regular breathing 10–15 s. Hypoxia was mild, but administration of enriched oxygen did stop periodic breathing. Elevation of inspired CO2 also prevented periodic breathing. The method used involved rebreathing which required a balance of flows into and out of a closed chamber and risk of possible hypoxia. It seems possible that inspired CO2 may have prevented apneas by raising breath CO2 above an apneic threshold. While such periodic breathing may not lead to problematic apneas in preterm infants, similar instability and significantly longer apneas occur in adults with obstructive sleep apnea (Salloum et al., 2010). Such adult patients also show a significantly changed apneic CO2 threshold compared to normals. Important differences also exist between infants and adults which will be discussed later. Models of ventilatory control have been previously applied to try to better understand such instability results, but with limited success possibly because of only applying linear control theory (Edwards et al., 2018). Loop gain has been measured in infants and tied to instability, but by itself cannot explain continuous periodic breathing. Apneic threshold is a highly nonlinear phenomenon, yet apneic threshold to CO2 is felt to be an important determinant of infant apneas during sleep (Gerhardt & Baricalari, 1984). Linear control theory can explain periodic breathing when loop gain is unity or larger at the oscillation frequency and phase shift led to positive feedback. Loop gain represents the product of chemoreceptor and plant gains. Plant gain includes the effects of cardiac output and lung volume. However, previous estimates of loop gain in infants did not report phase shift and continuous oscillation following sighs was not demonstrated suggesting negative feedback dominated (Edwards et al., 2018). Linear theory predicted ventilatory responses following sighs in infants could not predict any apneic pause prior to initiation of oscillations which significantly damped out within a minute (Fleming et al., 1984). A nonlinear model has been proposed for adults and infants which did predict apneas (Batzel & Tran, 2000). However, the parameters used were inconsistent with measured CO2 sensitivity and apneic threshold in preterm infants (Gerhardt & Baricalari, 1984). Periodic breathing was also tied to arousal from sleep which can be questioned (Katz, 2012). Recent work dealing with adults concluded that variable circulatory time delay to chemoreceptors can occur due to CO2 induced changes in brain blood flow and vascular volume (Yamashiro & Kato, 2021). Instability was predicted to follow periods of hyperventilation from a normocapnic baseline. Parallel changes of brain blood flow and brain vascular volume has been reported to occur in infants (Pryds et al., 1990). The question asked is whether inclusion of measured apneic threshold, chemosensitivity, and variable time delay can lead to model predicted periodic breathing without requiring abnormal chemosensitivity or an arousal response during sleep state transitions (Batzel & Tran, 2000). A nonlinear model incorporating an apneic threshold is proposed below which explores the stability implications. Infants, especially shortly after birth, sleep most of the time and sigh frequently as 0.9 sighs per minute (Fleming et al., 1984). Predicted responses to sighs during sleep were used as the disturbance to stimulate a dynamic response. If periodic breathing is sustained for more than 1/0.9 = 1.1 min following each sigh then continuous periodic breathing can be explained.

## 2 METHODS

**FIGURE 1:**
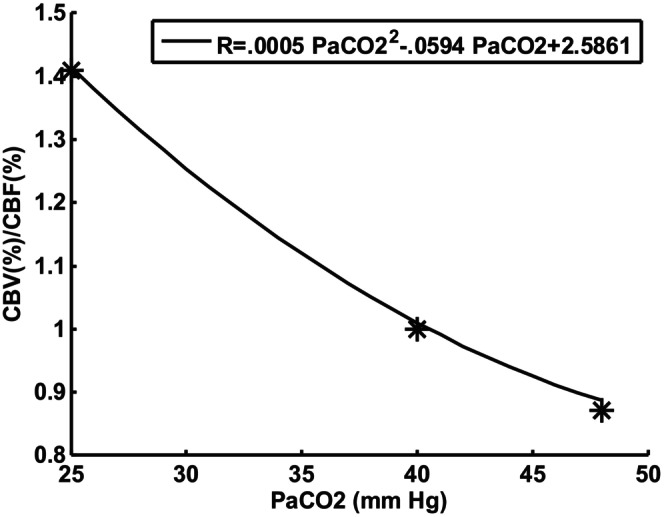
Quadratic fit of ratio CBV/CBF in adult humans (Fleming et al., 1984) as an index of circulatory delay to peripheral chemoreceptors.

The model was based on a previous model developed for adults during sleep but modified for infant application (Yamashiro, 2007). The main changes involved scaling for body weight, and apnea threshold. Apnea threshold description was based on measured CO2 responses in preterm infants with apnea episodes (Gerhardt & Baricalari, 1984). Dead space was also measured, and alveolar ventilation calculated by subtracting dead space ventilation. Alveolar ventilation is plotted versus PACO2 in Figure 1. Note that the apneic threshold for alveolar ventilation was 40.6 mmHg. The change in total and alveolar ventilation was assumed equal. In adults, this assumption is supported by a regression slope of 0.91 in normal subjects during CO2 inhalation and exercise (Gray et al., 1956). The apneic threshold was 4.8 mmHg below the baseline PACO2. Gerhardt and Baricalari (1984) also reported a different apneic threshold for a group without apnea episodes which was not considered here because of the focus on periodic breathing. The baseline CO2 level was significantly lower and CO2 sensitivity higher. Central and peripheral chemoreceptor responses to CO2 were based on first order differential equations found to provide valid dynamic fits in adults both intact and peripheral chemoreceptor denervated.

### 2.1 Chemoreceptors

(1) $$\;\frac{\text{dVac}}{\mathrm{dt}}=\left(G_{c}\times \left(P_{\text{ACO2}}\;\left(t-T_{d}\right)-P_{\mathrm{th}}\right)-V_{\mathrm{ac}}\right)/T_{c}$$

(2) $$\frac{\;\text{dVap}\;}{\mathrm{dt}}=\left(G_{p}\times \left(P_{\text{ACO2}}\;\left(t-T_{d}\right)-P_{\mathrm{th}}\right)-V_{\mathrm{ap}}\right)/T_{p}$$

(3) $$V_{a}=V_{\mathrm{ac}}+V_{\mathrm{ap}}$$

### 2.2 Lung and body tissue

(4) $$\begin{array}{c} K_{L}\times \frac{\text{dFACO}2}{\mathrm{dt}}=F_{\text{ICO2}}\times V_{a}+8.63\times Q\\ \;\times \left(C_{\text{TCO2}}-C_{\text{aCO2}}\right)/\left(B-47\right)-F_{\text{ACO2}}\times V_{a} \end{array}$$

(5) $$10\times K_{T}\times \frac{\text{dCTCO}2}{\mathrm{dt}}={\mathrm{MR}}_{\text{CO2}}+10\times Q\times \left(C_{\text{aCO2}}-C_{\text{TCO2}}\right)$$

where V_ac_ = central alveolar ventilation mL/min. V_ap_ = peripheral alveolar ventilation. G_c_ = central chemoreceptor gain mL/(min × mmHg). G_p_ = peripheral chemoreceptor gain. T_d_ = circulatory time delay to chemoreceptors min. T_c_ = central time constant min. T_p_ = peripheral time constant min. K_L_ = lung volume − functional residual volume (FRC) liters. K_T_ = tissue volume − total body water (TBW) liters.

### 2.3 Circulatory time delay

Circulatory time delay T_d_ was assumed to be determined by the ratio of brain vascular volume V_b_ and brain blood flow Q_b_.

(6) $$T_{d}=\mathrm{Vb}/\mathrm{Qb}$$

Fortune et al. (1995) measured cerebral blood flow V_b_ and blood volume Q_b_ in eight normal human adults for three PaCO2 levels (25, 40, and 48 mmHg). The ratio V_b_/Q_b_ after dividing by the value at P_aCO2_ = 40 can be fitted with a quadratic as shown in Figure 1. Pryds et al. (1990) attempted a similar measurement in 1–2 day old preterm infants, but V_b_ could not be normalized in the same way due to a variable imaging path length. However, Pryds et al. showed that both V_b_ and Q_b_ changed in parallel in response to P_ACO2_ in infants similar to the adult results. Both V_b_ and Q_b_ vary in adults and infants in proportion to body weight so the ratio could be independent of size. This ratio was multiplied by a constant to adjust the time delay at P_acO2_ = 40 to what has been experimentally measured by transient response to inhaled CO2 (Sovik & Lossius, 2004) in infants. The effect of P_ACO2_ on V_b_ in 1–2‐day old infants (Pryds et al., 1990) appeared to be different in comparison to adults and will be considered. Such a difference was expected due to the difference in amount of vascular smooth muscle cells and low brain blood flow autoregulation in preterm infants (Haruda, 2001).

### 2.4 Inhaled CO2 response

**FIGURE 2:**
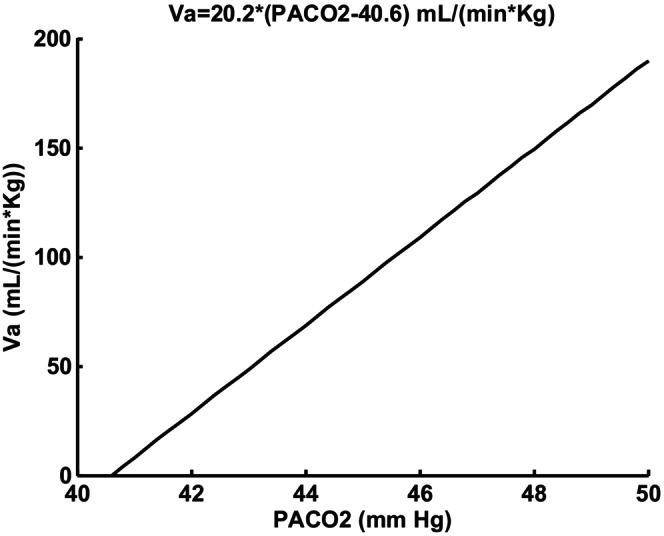
Average CO2 response curve in preterm infants (Fortune et al., 1995) with apnea.

**FIGURE 3:**
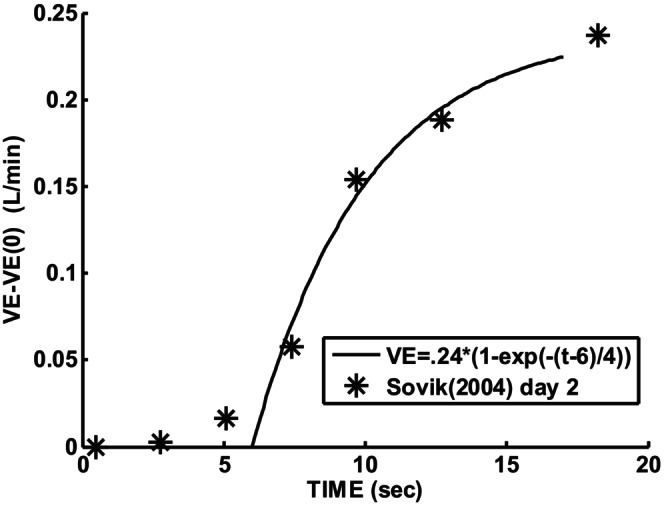
Preterm 2‐day infant transient ventilatory response to inhalation of 3% CO2. Responses averaged over 26 infants (Kattwinkel et al., 1975). Solid curve was fitted visually to the data.

**FIGURE 4:**
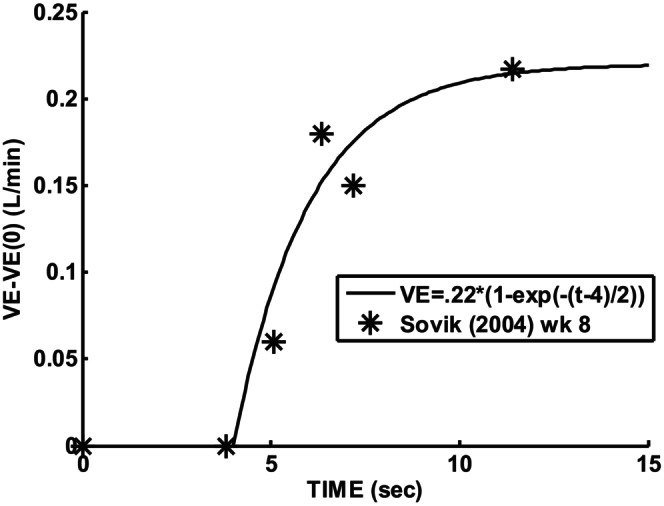
Preterm 8‐week infant transient ventilatory response to inhalation of 3% CO2.

Gerhardt and Baricalari (1984) reported that preterm infants who exhibited significant apneas had inhaled CO2 responses which corresponded to what is reproduced in Figure 2. Note that apnea (V_a_ = 0) is predicted if P_ACO2_ falls below 40.6 mmHg. The slope of the response curve of 20.2 mL/(min × mmHg × Kg) had a standard deviation leading to a 52% higher value. Thus, a slope range 22 to 33 mL/(min × mmHg) for an average body weight of 1.07 Kg was consistent with their results. This slope corresponds to combined central and peripheral responses. The baseline or control P_aCO2_ was hypercapnic at 45.4 mmHg. The upper value of 33 was used to explore possible instability. Sovik and Lossius (2004) studied transient responses to 15 s of inhaled CO2 in term infants which allowed dynamic separation of peripheral (1–3 s time constant) and central (60–70 s time constant). Averaged transient responses for 8 weeks old infants showed an initial (peripheral) sensitivity of 220 mL/(min × mmHg) following an estimated delay of 4 s as shown in Figure 3. A minimum reported body weight threshold of 2.5 Kg and a measured end‐tidal P_etco2_ change of 6.5 mmHg at 2 s leads to 14.5 mL/(min × mmHg) estimate for a 1.07 Kg infant. There is also a difference in metabolic rate between term and preterm infants which occurs even after dividing by body weight (Dechert et al., 1985). Assuming that alveolar ventilation must be directly proportional to metabolism, an additional metabolic multiplying factor of 1.8 is justified which is the measured ratio of preterm to term CO2 production rates (Dechert et al., 1985) resulting in 26 mL/(min × mmHg). This was the justification for initial setting of model G_p_ = 26, G_c_ = 7 mL/(min × mmHg), T_p_ = 2, T_c_ = 60, and T_d_ = 4 s. The measured transient response for 2 day olds is shown in Figure 4. Using the same rationale as detailed for 8 week infants leads to: G_p_ = 24, G_c_ = 9 mL/(min × mmHg), T_p_ = 4, T_c_ = 60, and T_d_ = 6 s.

### 2.5 Endogenous CO2 response

Rigatto et al. (1991) reported that in preterm infants ventilatory responses to endogenous P_CO2_ on average led to 76% higher response gain than what was measured for inhaled CO2 in the steady state. Such a difference has not been observed in resting adults except for extreme hypercapnia where narcotic effects can occur (Eisele et al., 1967). Endogenous responses were estimated from peak‐to‐peak changes in breath‐by‐breath ventilation and end‐tidal P_CO2_ during periodic breathing. Even transient responses to inhaled CO2 conducted similarly to Sovik and Lossius (2004) led to CO2 response gains higher than steady state but less than endogenous estimates. These estimates should mainly reflect peripheral chemosensitivity which exceed steady state sensitivity due to suspected prolonged hypercapnia depression. Model predictions were also made based on these results which suggested that most prior estimates of peripheral chemosensitivity in preterm infants significantly underestimated the value applicable to normal breathing. The reported endogenous sensitivity was 0.067 L/min/(mmHg × Kg) which for a 1.07 Kg body weight justified G_p_ = 0.072 L/min/mmHg.

The predictions based on inhaled and endogenous CO2 were separately made and compared in the discussion section.

Normal cardiac output for preterm infants was assumed to be 221 mL/(min × Kg) (Alverson et al., 1984) so 236.5 mL/min for 1.07 Kg body weight. Normal lung volume (functional residual capacity (FRC)) was assumed to be 21.6 mL/Kg or 23.1 mL for 1.07 Kg body weight (Latzin et al., 2009). Other parameters were assumed to scale as a function of body weight and estimated values for infants or adults were used. A spontaneous deep breath was simulated as an alveolar ventilation of 0.3 L/min for 3 s.

The model differential equations were solved numerically using Matlab function ode45. Predictions of mainly minute ventilation versus time were made from separate runs for different parameter values.

## 3 RESULTS

### 3.1 Peripheral gain estimated using external CO2 response

**FIGURE 5:**
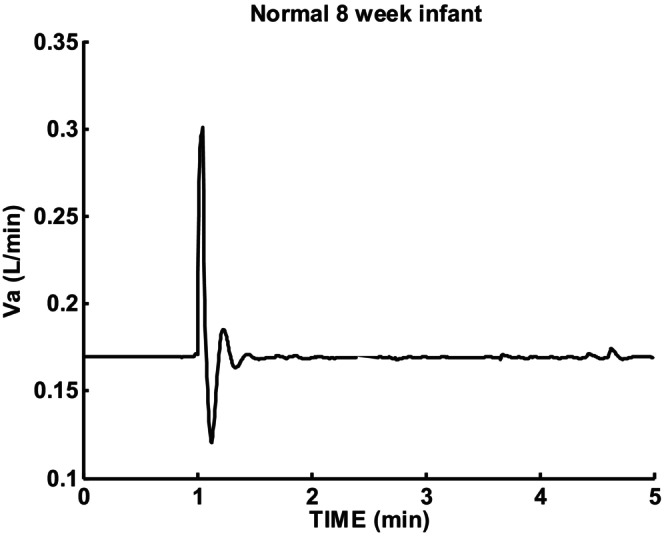
Sigh response for normal 8‐week infant. *β* = −0.29, T = 12.9 s, ζ = 0.6.

The predicted ventilation response to a sigh is shown in Figure 5 for within normal range of all parameters using week 8 infant parameters. The response shows an initial decrease followed by a slight overshoot and return to baseline. No periodic breathing or significant damping was observed and the damping ratio ζ = 0.6 was close to the critical damping value of 0.7. *β* = −ζωr = damping factor as defined by Fleming et al. (1984). Damping ratio ζ corresponds to a linear second order differential equation parameter which is related to the % overshoot (PO) as:

(7) $$\zeta =\frac{-\ln\left(\frac{\mathrm{PO}}{100}\right)}{{\left(\pi^{2}+\ln^{2}\left(\frac{\mathrm{PO}}{100}\right)\right)}^{1/2}}$$

T = oscillation period in seconds, which some have referred to as oscillation duration can be estimated from the measured time between two peaks.

**FIGURE 6:**
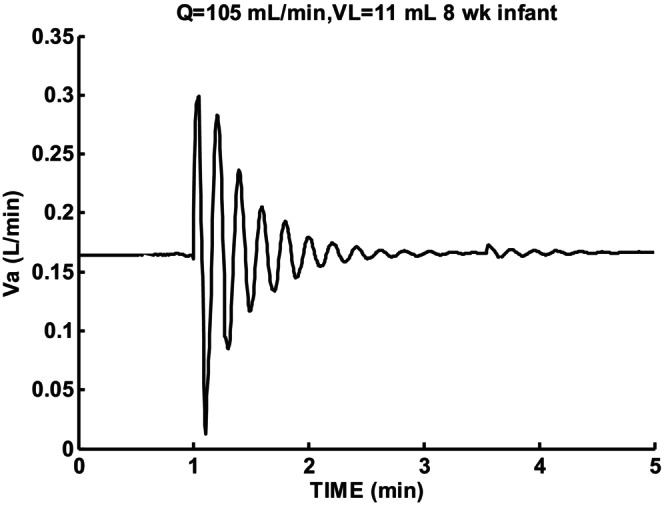
Sigh response for 8‐week infant for low cardiac output Q and lung volume VL. Initial following cycle with T = 11.4 s and subsequent T = 13.2 s. During damped oscillation *β* = −0.124 and ζ = 0.26.

The parameters estimated as shown in Figures 3 and 4 were used as constraints and not adjusted. This left cardiac output and lung volume as the variables explored for stability effects. Ventilatory sigh response for low cardiac output (105 mL/min) and lung volume (11 mL) is shown in Figure 6. One cycle of periodic breathing was predicted followed by a damped oscillatory second order response. Responses were sensitive to parameter choices which were selected in this case to illustrate combined nonlinear and linear characteristics. Apneic threshold was reached for one cycle and led to an asymmetrical ventilation pattern. Note that the oscillation period T = 11.4 s when apneic threshold was reached was shorter than the 13.2 s measured during damped oscillation. This response pattern shows that nonlinear oscillation can initially occur followed by a damped linear oscillation return to control level. Oscillation epochs are then possible without satisfying linear theory oscillation criteria.

**FIGURE 7:**
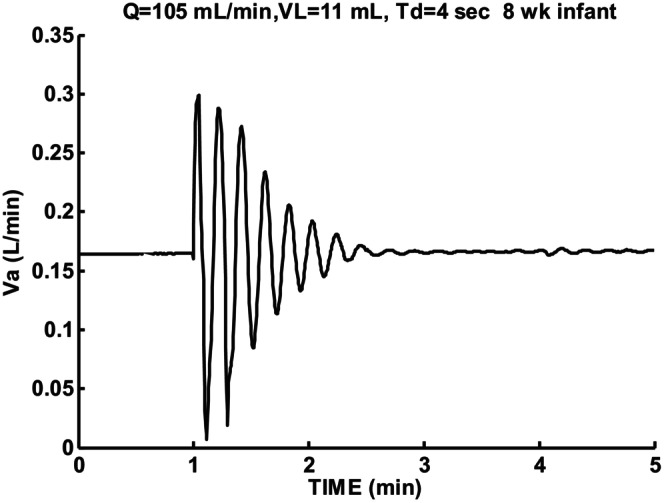
Sigh response for 8‐week infant for low cardiac output Q and lung volume VL with Td = constant 4 s. Initial following cycle with T = 12.3 s and subsequent T = 12 s. During damped oscillation *β* = −0.0732 and ζ = 0.14.

Figure 7 shows sigh response for the same cardiac output and lung volume as Figure 6 except circulatory delay Td was kept constant at 4 s rather than change with P_CO2_. Compared to Figure 6 instability increased slightly. The calculated damping coefficient decreased from 0.26 to 0.14 indicating increased instability. The baseline P_CO2_ of 45.5 mmHg was higher than the normal level of 40 mmHg (45.4 mmHg reported by Sovik and Lossius (2004)) so average variable delay could be estimated as 3.8 rather than 4 s according to Figure 1. This difference was not sufficient to account for this change. Variable time delay due to P_CO2_ then led to reduced instability.

**FIGURE 8:**
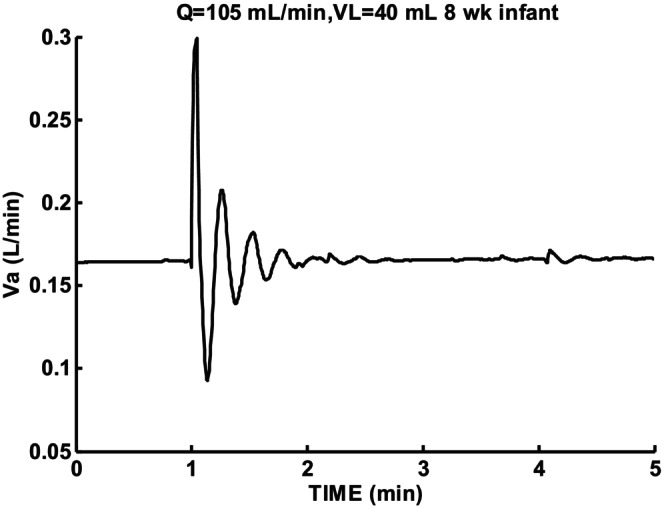
Sigh response for 8‐week infant for low cardiac output Q = 105 mL/min and increased lung volume VL = 40 mL. Damped oscillation *β* = −0.157, T = 16.8 s, and ζ = 0.405.

Increasing lung volume to a larger than normal value of 40 mL eliminated any periodic breathing and increased damping and oscillation period as shown in Figure 8. A damped oscillation response resulted with a longer period of 16.8 s compared to a period of 13.2 s for Figure 6. Linear theory appears adequate to describe this response. Increasing lung volume was then a possible way to stop periodic breathing and reduce instability.

**FIGURE 9:**
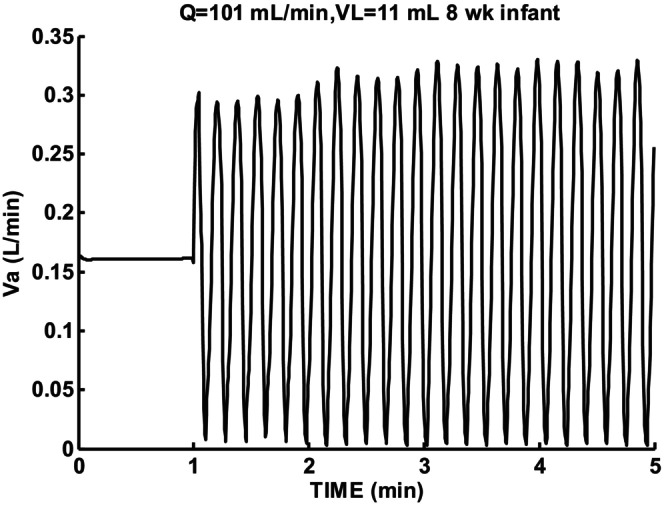
Sigh response for 8‐week infant for low cardiac output Q = 101 mL/min and low lung volume VL = 11 mL. Oscillation period T = 11.4 s.

Figure 9 shows sigh response for a slightly lower cardiac output Q = 101 mL/min and low lung volume VL = 11 mL. Periodic breathing with an epoch duration longer than 5 min resulted.

**FIGURE 10:**
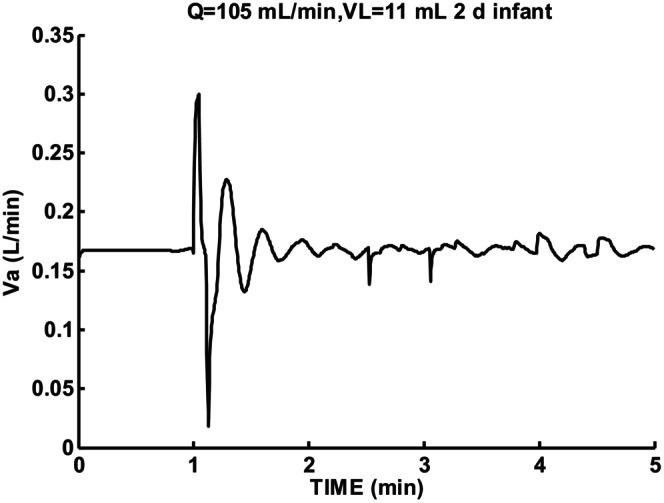
Sigh response for 2‐day infant for low cardiac output Q = 105 mL/min and lung volume VL = 11 mL. Damped oscillation *β* = −0.104, T = 19.2 s, and ζ = 0.318.

Figure 10 shows sigh response using parameters for 2‐day infant. Cardiac output and lung volume were set to the same values as 8‐week infant listed in Figure 6. No periodic breathing epoch resulted and increased oscillation period T = 19.2 s and damping coefficient ζ = 0.318. Two‐day infant responses showed less instability compared to 8‐week infant.

### 3.2 Peripheral gain estimated using endogenous CO2 response

**FIGURE 11:**
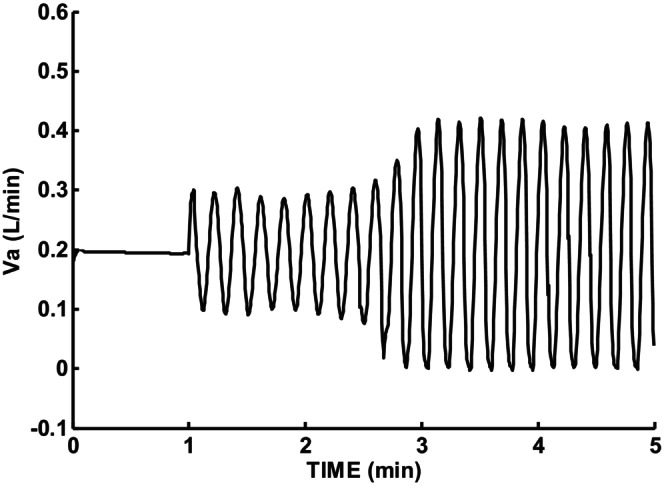
Sigh response with gain set from endogenous CO2 measurement (Rigatto). Gp = 0.072 VL = 0.02311 L, T = 0.18 min (10.5 s) near 5 min, T = 11.7 s near 2 min.

**FIGURE 12:**
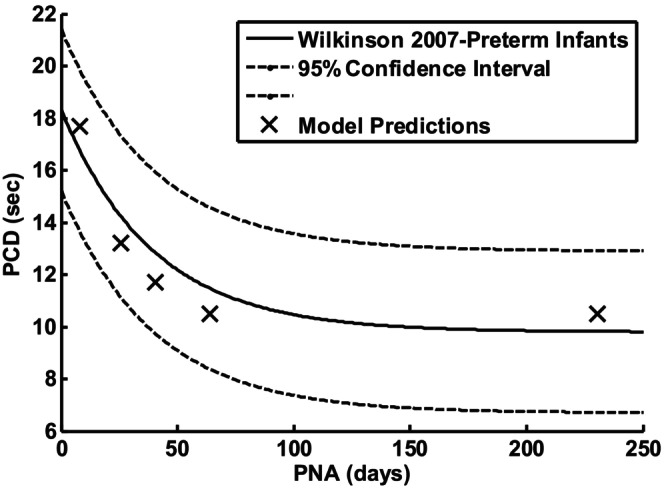
Periodic breathing cycle duration PCD plotted versus postnatal age PNA. Solid and dashed lines (95% confidence interval (Wilkinson et al., 2007)). Model predictions indicated by X symbol.

Figure 11 shows model predicted sigh response using peripheral gain G_p_ = 0.072 L/min/mmHg as estimated by Rigatto et al. (1991) based on endogenous responses in preterm infants. Both cardiac output and lung volume were set at normal values. Sustained periodic breathing was predicted with a periodic cycle duration (PCD) initially at minute 2 of 11.7 s and at minute 5 was 10.5 s. Figure 12 shows model predicted sigh response when peripheral gain was kept the same, but lung volume VL was doubled (2 × 0.02311 L). The increase in lung volume was sufficient to dampen out periodic breathing within 2 min.

## 4 CONCLUSION

Spontaneous deep breaths can initiate an initial apneic period followed by an unstable recovery which can take several minutes. Predicted responses were constrained by experimentally measured chemoreceptor parameters. When cardiac output and lung volume were set to normal values periodic breathing was not predicted and linear theory can be used to describe responses. Combined low cardiac output and lung volume was predicted to be able to lead to a periodic breathing epoch following a deep breath for a long enough duration for another deep breath to occur. In this way spontaneous deep breaths could lead to sustained periodic breathing. The destabilizing effect of low cardiac output and lung volume can be reduced by increased lung volume. Lung volume increase was then predicted to be an effective means of stabilizing periodic breathing. The change in circulatory delay due to CO2 was also a stabilizing factor.

Incorporating higher peripheral chemoreceptor gain as measured during periodic breathing and endogenous P_CO2_ led to model predicted continuous periodic breathing even with normal values of cardiac output and lung volume.

## 5 DISCUSSION

### 5.1 Inhaled CO2 response

The current model and simulation results found that the main determinants of respiratory stability were peripheral chemoreceptor gain, cardiac output, and lung volume. When these parameters were set at normal values no periodic breathing was predicted. Chemoreceptor gain when increased above normal is known to lead to predicted instability. Such an increase in loop gain has not been shown to occur in infants (Edwards et al., 2018). Chemoreceptor gains set in the normal range combined with low cardiac output and lung volume led to a predicted epoch duration of periodic breathing by the present model. The duration of periodic breathing epochs predicted can exceed the expected time between sighs. Periodic breathing could then be continued without interruption. This type of instability disappeared when lung volume was set at a larger than normal value. This is an important finding as it explains the mechanism by which increasing lung volumes using nasal continuous positive airway pressure (CPAP), reduces the frequency of apnea and periodic breathing in preterm infants (Kattwinkel et al., 1975).

A constant circulatory delay was found to lead to increased instability compared to circulatory delay varying with arterial blood P_CO2_. Conditions following a sigh varies from hypocapnia to hypercapnia in the lungs. Hypercapnia changes blood flow and blood volume and the ratio determines circulatory delay. The estimated ratio is shown in Figure 1. If the effect of blood flow dominates, then hypercapnia should increase brain blood flow and decrease circulatory delay. The average P_CO2_ measured over the minute following the sigh shown in Figure 6 was 0.23 mmHg higher than baseline. This shift was caused by an asymmetric cycling of P_CO2_ which favored an increase. Increased brain blood flow and decreased circulatory delay was then predicted. Decreasing control loop phase shift helps stability. If the effect of vascular blood volume dominated the opposite effect of instability would be possible. The balance between cerebral blood flow and blood volume has not received much attention previously, but the current simulation results suggest potentially important roles in abnormal cases.

Periodic breathing in preterm infants occurs with very high prevalence (Fenner et al., 1973). However, it has not been considered problematic since it does not lead to long apneic durations and significant hypoxia. Periodic breathing has then been observed but left largely untreated. This may be one reason why infants born preterm are more likely to develop obstructive sleep apnea (OSA)when school‐aged (Tapia et al., 2016). In adults, centrally mediated respiratory instability is strongly linked to OSA (Younes et al., 2001). The development of periodic breathing in preterm infants may predispose them for later OSA.

Wilkinson et al. (2007) compared periodic breathing in term and preterm infants. They reported that periodic breathing cycle duration (PCD) in preterm infants decreased on average from 18.3 to 9.8 s with age. Figure 10 (19.2 s‐day 2) and 6 (11.4–13.2 s‐week 8) were consistent with their results. Their postulated reduced chemoreceptor delays as the cause was supported by using the published responses of Sovik (Sovik & Lossius, 2004).

Sigh frequency is high in younger infants (Fleming et al., 1984). The reasons for this are complex (Ramirez et al., 2022) and include reflex responses to chemoreceptor stimulation and lung atelectasis. The supine rather than prone position was postulated to decrease sighs in infants. Another discussed connection to sighs was arousal. In the present model the CO2 response curve corresponded to measurements made on infants with apnea. The baseline CO2 level was significantly higher than the group without apnea and closer to the apneic threshold. Difference in sleeping posture (prone or supine) and CO2 may have caused a difference in sigh frequency and repeated apneic responses. Periodic breathing can then be promoted by increased sigh frequency.

The current model predictions unlike earlier simulations predicted continuous periodic breathing in preterm infants based on measured parameters without resorting to assumed effects of arousal or abnormal chemoreceptor gains. Chemoreceptor gains were considered a constraint based on previously measured values in this study, but can also significantly affect stability. Sighs are known to occur frequently in younger infants and lead to subsequent periods of apnea. Normal measured parameters led to predicted highly damped subsequent ventilatory response. Combined low cardiac output and lung volume led to predicted periodic breathing for a duration long enough to expect another sigh to occur. Epochs of periodic breathing were tied to nonlinear apneic threshold to CO2 and could not be explained by linear control theory. Increased lung volume was predicted to be highly effective in preventing periodic breathing.

### 5.2 Endogenous CO2 response

The endogenous CO2 estimates of chemosensitivity as pioneered by Rigatto et al. (1991) was used by the current model to successfully predict continuous periodic breathing under conditions of normal cardiac output and lung volume. As noted above, the period of periodic breathing in preterm infants is only 10.5 s. The response to such a short period will mainly be due to the peripheral chemoreceptors which we estimated to have a time constant of 2 s for preterm infants. The transient response this was based on was 15 s long. Thus, the peak‐to‐peak fluctuations of ventilation and end‐tidal CO2 occur with this period and their ratio reflect mainly peripheral chemoreceptor responses. From the simulation run for Figure 11 the peak‐to‐peak ventilation was 0.42 L/min. The corresponding alveolar CO2 peak‐to‐peak value was 9 mmHg. The ratio was 0.05 L/min/mmHg which was 70% of the G_p_ value used. A 30% underestimate of G_p_ was predicted using the endogenous value. Repeating the simulation with this higher G_p_ value did not significantly change Figure 11 predictions and was not included. The main contribution of using endogenous estimates of chemosensitivity was being able to predict continuous periodic breathing using chemosensitivity parameters measured in preterm infants and normal values for cardiac output and lung volume.

PCD decreases as a function of postnatal age (PNA) according to an exponential equation (Wilkinson et al., 2007). Figure 11 was based on endogenous estimates of peripheral chemosensitivity and predicted a 10.5 s periodic breathing cycle duration which was within the reported range. The exponential age dependence was found to be quite robust and applied to both preterm and term infants. Franz et al. (1976) reported CO2 response sensitivities in preterm infants at PNA of 3 days of 10.8 mL/(min × Kg) and 53.5 mL/(min × Kg) at 33 weeks (231 days). Assuming the same PNA dependence as PCD, with constants chosen to fit the measured G values led to the following estimate.

(8) $$G=53.63-46.23\times \exp\;\left(-0.0255\times \mathrm{PNA}\right)$$

where PNA = postnatal age in days, and the PNA multiplier is from (Sovik & Lossius, 2004).

The above gain was used to scale G_p_ so G_p_ = G × 72/53.5 = 72 mL/(min × mmHg). Then model simulations were repeated exactly as for Figure 11 except for _Gp_ = 40, 50, and 60 mL/(min × mmHg). The corresponding PNA values were then 25.9, 40.5, and 64.08 days. The predicted PCD values as a function of PNA are shown in Figure 12. Note that the PCD prediction at a PNA of 231 days was based on the reported PNA and sensitivity. The solid curve can be considered as measured PCD so the agreement between model predicted and this reference curve serves as model validation as well as support for the assumed exponential change of gain G with PNA.

Model predictions for G_p_ less than 40 corresponding to PNA below 10 days led to a highly damped response following a sigh with no detectable oscillation. This motivated consideration of the exaggerated effect noted earlier of P_ACO2_ on brain vascular volume V_b_ in 1–2 day old preterm infants (Pryds et al., 1990). The following two equations were based on their reported results:

CBF = 10.1 + 0.368 × (P_ACO2_−33).

CBV = 1.7 + 0.0861 × (P_ACO2_−33).

**FIGURE 13:**
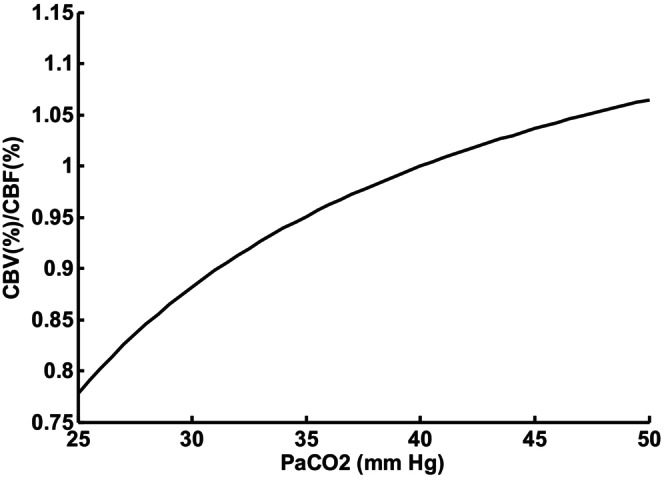
CBV/CBF in 1–2 day old preterm infants (Pryds et al., 1990) as an index of circulatory delay to peripheral chemoreceptors normalized to the value at PaCO2 = 40 mmHg. Predicted PCD in Figure 12 for PNA = 7.8 days and Gp = 25 mL/(min × mmHg) used this curve to predict circulatory time delay.

The ratio CBV/CBF was used to estimate circulatory time delay and was normalized to estimate a measured time delay at P_ACO2_ = 40 mmHg as described earlier. This ratio is plotted versus P_ACO2_ in Figure 13. Note that an increased time delay was predicted at P_ACO2_ > 40 mmHg in contrast to the decrease shown in Figure 1 which was based on adult measurements. This time delay increase decreases stability and permitted estimation of PCD for G_p_ = 25 mL/(min × mmHg), PNA = 7.8 days, PCD = 17.7 s which was also plotted in Figure 12.

The diverse model predictions depending on how CO2 sensitivity and circulatory time delay were estimated demonstrated how labile infant responses can be. This point was previously made by Rigatto et al. (1991) who emphasized the importance of using endogenous CO2 to assess normal sensitivity. The predictions based on endogenous CO2 should then be the most relevant for normal preterm infants as Figure 13 suggested. Externally applied CO2 gain estimates were found to predict damped responses except for low cardiac output and lung volume. Even the periodic breathing predicted under these extremes could be stabilized by increased lung volume. Increased lung volume is then the simplest method to stop periodic breathing. The effect of P_ACO2_ on brain vascular blood volume Vb appears to also be very different in the very young and changes cerebral circulatory time delay from prolongation to shortening with maturation. Circulatory delay and not loop gain appears to be the cause of periodic breathing in very young preterm infants. High incidence of periodic breathing in very young preterm infants may then be caused by immature development of brain vascular response to CO2.

## AUTHOR CONTRIBUTIONS

S.Y. and N.I. conception and model development. S.Y. drafted manuscript. S.Y. edited and revised manuscript. S.Y. and N.I. approved final version of manuscript.

## FUNDING INFORMATION

None.

## CONFLICT OF INTEREST STATEMENT

No conflicts of interest, financial or otherwise, are declared by the author(s).

## ETHICS STATEMENT

The human infant data used (all pre 2008) were reported to be in accordance with the ethical standards of the responsible committee on human experimentation (institutional and national) and with the Helsinki Declaration of 1975.

## Data Availability

All data used were not collected by the authors and sources have been referenced.
